# Supplementary material for: Herpes Simplex Virus Re-Activation in Patients with SARS-CoV-2 Pneumonia: A Prospective, Observational Study
Source: Microorganisms. 2021 Sep 7;9(9):1896. doi: 10.3390/microorganisms9091896 (PMC8465957; doi:10.3390/microorganisms9091896)

Technical specifications

HSV-1 PCR.

Serum HSV-1-DNA was detected and quantified by HSV-1 ELITe MGB real-time polymerase chain reaction of ELITech Group with a threshold of sensitivity for detection of 140 copies/mL and a linearity range from 140 copies/mL to 14,000,000 copies/mL. HSV-1-DNA on respiratory samples was detected and quantified by HSV-1 ELITe MGB real-time polymerase chain reaction of ELITech Group with a threshold of sensivity for detection of 144 copies/mL and a linearity range from 144 copies/mL to 14,400,000 copies/mL. CPE-internal control (extraction and inhibition control) was used as internal control template for DNA extraction from clinical samples with the ELITechGroup S.p.A. products.

CMV PCR

Blood CMV-DNA was detected and quantified by the ABBOTT RealTime CMV assay of ABBOTT group, with a threshold of sensivity for detection of 62 UI/mL and a linearity range from 62 to 156,000,000 UI/mL. CMV-DNA on respiratory samples was detected and quantified by CMV ELITe MGB real-time polymerase chain reaction of ELITech Group with a threshold of sensivity for detection of 187 UI/mL and a linearity range from 187 to 18,700,000 UI/mL.

**Figure 1A:** Assumption for the underlying structure of the data for the model with use of tocilizumab as the key exposure

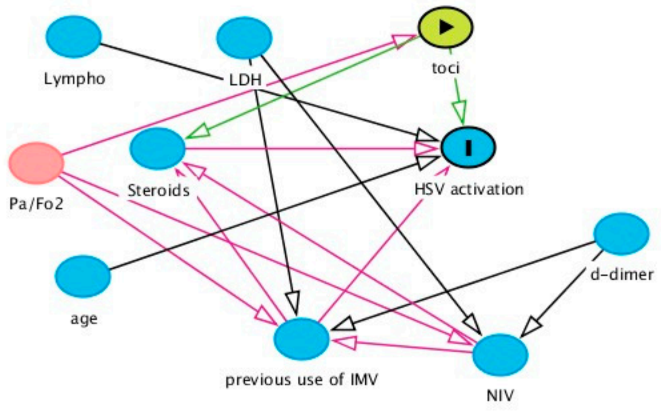


**Figure 1B:** Assumption for the underlying structure of the data for the model with use of steroids as the key exposure

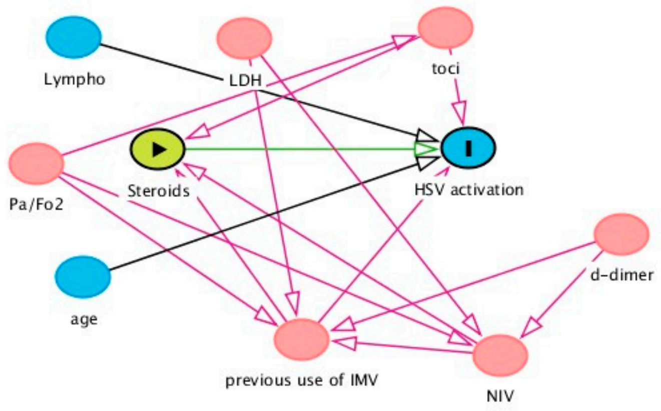

Supplement: Supplementary file 1 [file microorganisms-09-01896-s001.zip › microorganisms-1362358-supplementary.pdf]
